# Supplementary material for: 3D-Printed Polyamide 12/Styrene–Acrylic Copolymer–Boron Nitride (PA12/SA–BN) Composite with Macro and Micro Double Anisotropic Thermally Conductive Structures
Source: Polymers (Basel). 2023 Jun 22;15(13):2780. doi: 10.3390/polym15132780 (PMC10347135; doi:10.3390/polym15132780)
Supplement: Supplementary file 1 [file polymers-15-02780-s001.zip › polymers-2413039-supplementary.pdf]

3D-Printed Polyamide 12/Styrene–Acrylic Copolymer–Boron Nitride (PA12/SA–BN) Composite with Macro and Micro Double Anisotropic Thermally Conductive Structures

Minhang Chen [1,2,†], Xiaojie Chen [1,†], Junle Zhang [3], Bingfeng Xue [3], Shangyu Zhai [1], Haibo She [4], Yuancheng Zhang [1], Zhe Cui [1], Peng Fu [1], Xinchang Pang [1], Minying Liu [1] and Xiaomeng Zhang [1,2,4,*]

[1] School of Materials Science and Engineering, Henan Key Laboratory of Advanced Nylon Materials and Application, Engineering Laboratory of High-Performance Nylon Engineering Plastics of China Petroleum and Chemical Industry, Zhengzhou University, Zhengzhou 450000, China; lmy@zzu.edu.cn (M.L.)
[2] The State Key Laboratory of Polymer Materials Engineering, Polymer Research Institute, Sichuan University, Chengdu 610065, China
[3] Faculty of Engineering, Huanghe Science and Technology University, Zhengzhou 459000, China
[4] Jinguan Electric Co., Ltd., Nanyang 473000, China
* Correspondence: zhangxm@zzu.edu.cn
† These authors contributed equally to this work.

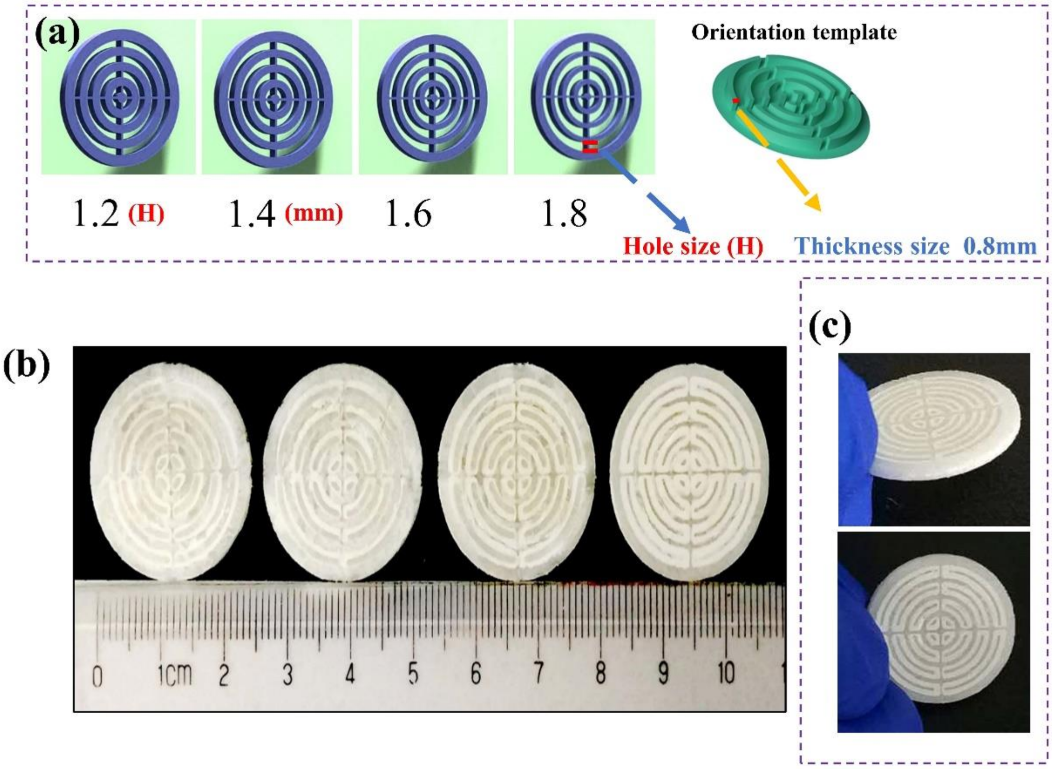

Figure S1. (a) Designed models of orientation template materials and template materials with different hole sizes, (b) final orientation template composites, and (c) stereoscopic images of the orientation template composite.

The matrix template materials with different hole sizes of 1.2, 1.4, 1.6 and 1.8 mm were prepared in this work, as shown in Figure S1. The contents of BN in the orientation template composite with the hole size of 1.2, 1.4, 1.6 and 1.8 mm were 7.8 wt%, 10.4 wt%, 15.9 wt% and 21.4 wt% respectively, and the calculation method was also presented in the supplementary material.

In order to investigate the effect of the oriented BN on the thermal conductivity of the composite, the template composite with the same content of BN as the orientation template composite were prepared, as shown in Figure 2. It should be mentioned that the total hole volumes of the orientation template composite and the template composite

are the same. Similarly, in order to investigate the effect of the macro and micro double anisotropic structures on the thermal conductivity of the composite, the isotropic samples (PA12/BN) with the same BN content (7.8 wt%, 10.4 wt%, 15.9 wt% and 21.4 wt%) were prepared by twin-screw extrusion and compression molding at 1 MPa and 200 °C. Furthermore, it should be clarified that the thermal conductivity of SA is similar to that of PA12, so the SA has little effect on the thermal conductivity of the composite. Therefore, the thermal conductivity of the final composite was mainly determined by the distribution and morphology of BN.

It should be mentioned that the calculation method of filler content in orientation template composite and template composite is as follows: First, the weight ratio between SA and BN is a constant ($\boldsymbol{\alpha}$) of 2:3, thus the BN content in SA-BN compound is 60 wt%. Then, the quality of SA-BN compound in the final composite can be easily obtained by the mass of the final template composite ($\boldsymbol{W_{end}}$) minus that of the 3D printed sample ($\boldsymbol{W_{3D}}$). Therefore, the total BN content in the final composite equals $\boldsymbol{\alpha(W_{end}-W_{3D})/W_{end}}$.

Table S1. Parameters applied in the 3D printing.

Parameters	Value
Powder supply cylinder temperature	150℃
Molding cylinder temperature	171.5℃
Laser power	30 W
Scan speed	2.28 m/s
Scan distance	0.08 mm
Layer thickness	0.1 mm

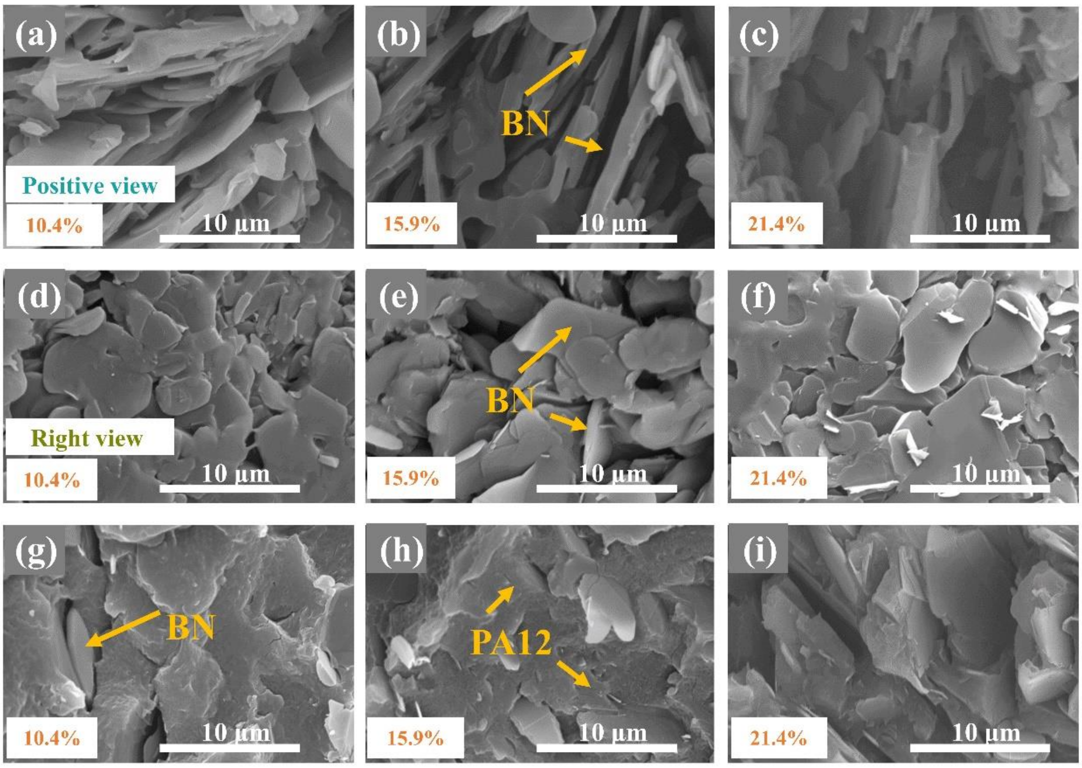

Figure S2. SEM images of (a-f) orientation template composite along different view directions and (g-i) isotropic composite.

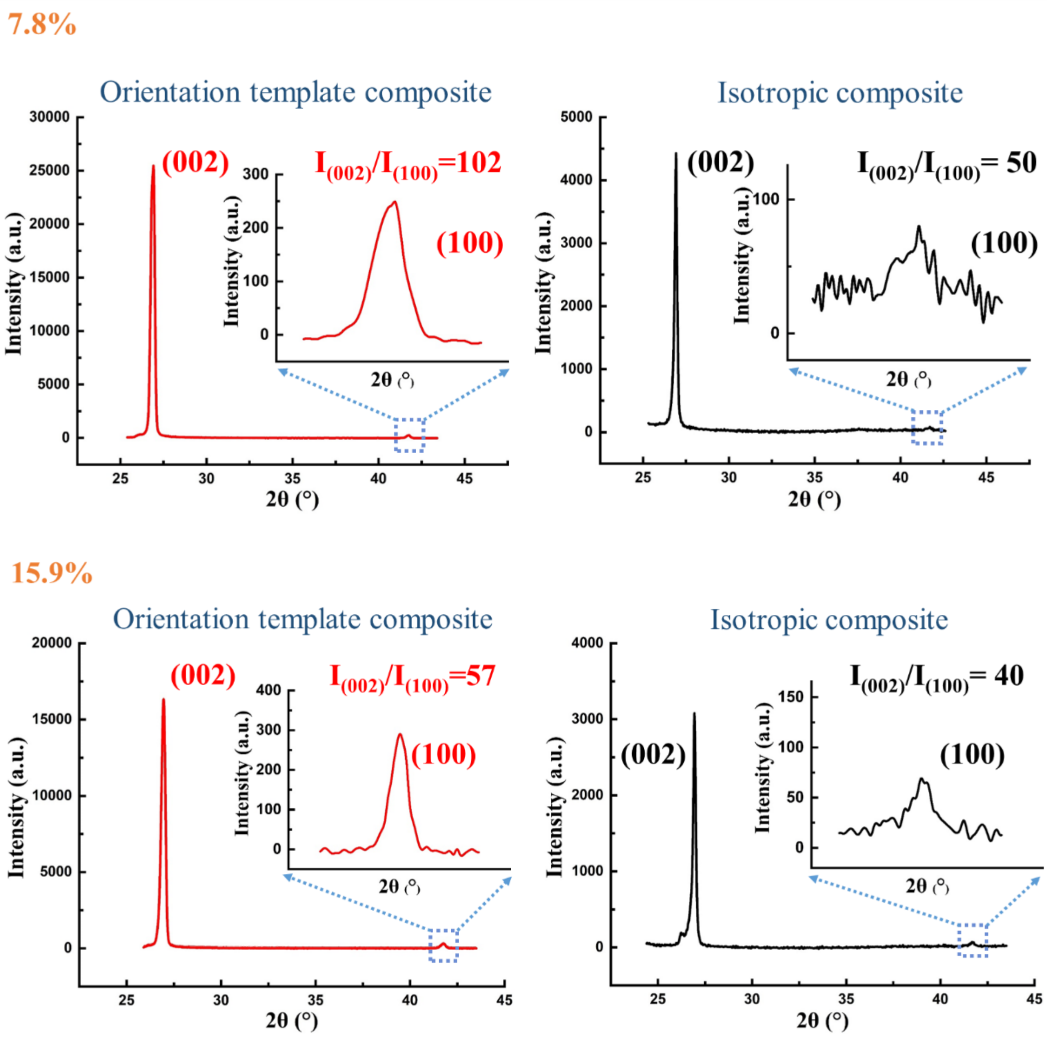

Figure S3. XRD patterns of the orientation template and isotropic composites with BN content of 7.8 wt% and 15.9 wt%.

Table S2. Major parameters used in simulation.

Parameters	PA12	SA	BN	
Thermal conductivity (W/(m·K))	0.36	0.2	In-plane	Through -plane
			600	30
Specific heat capacity (J/(kg·K))	1800	2000	710	
Density (kg/m^3)	950	1000	2250	
Uniform temperature (°C)	25	25	25	

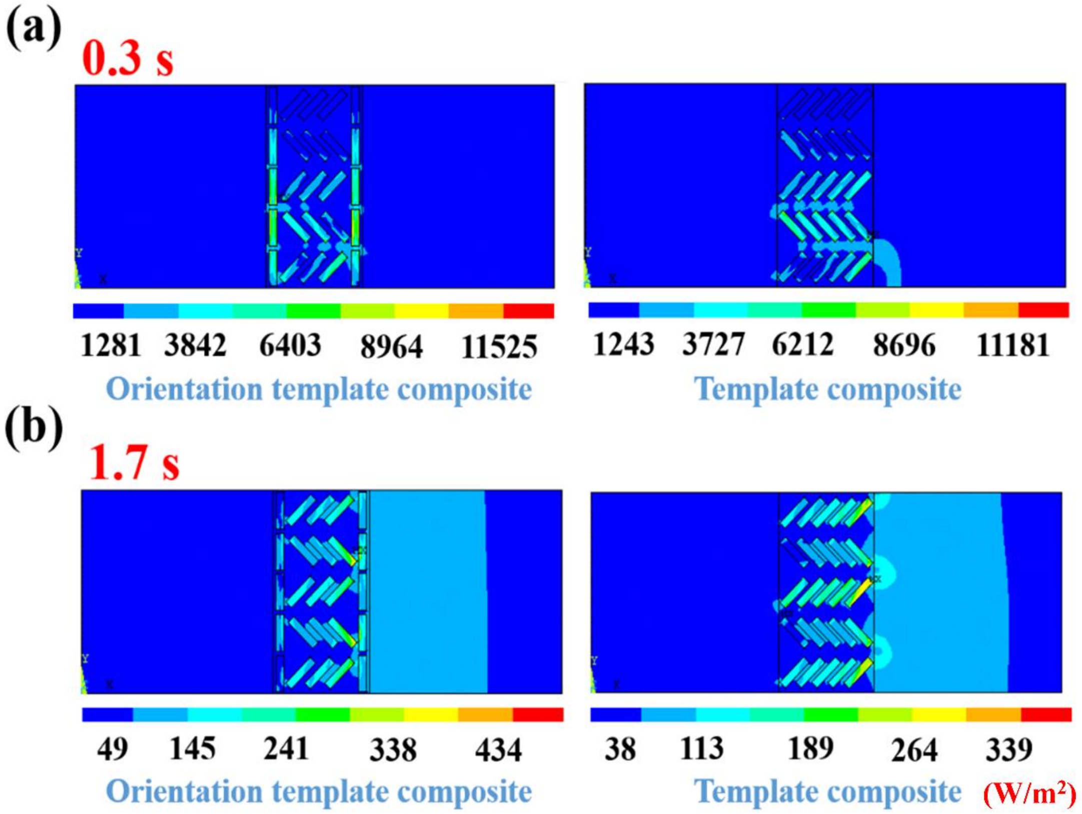

Figure S4. Sum of heat flux of different models at (a) 0.3 s and (b) 1.7 s.

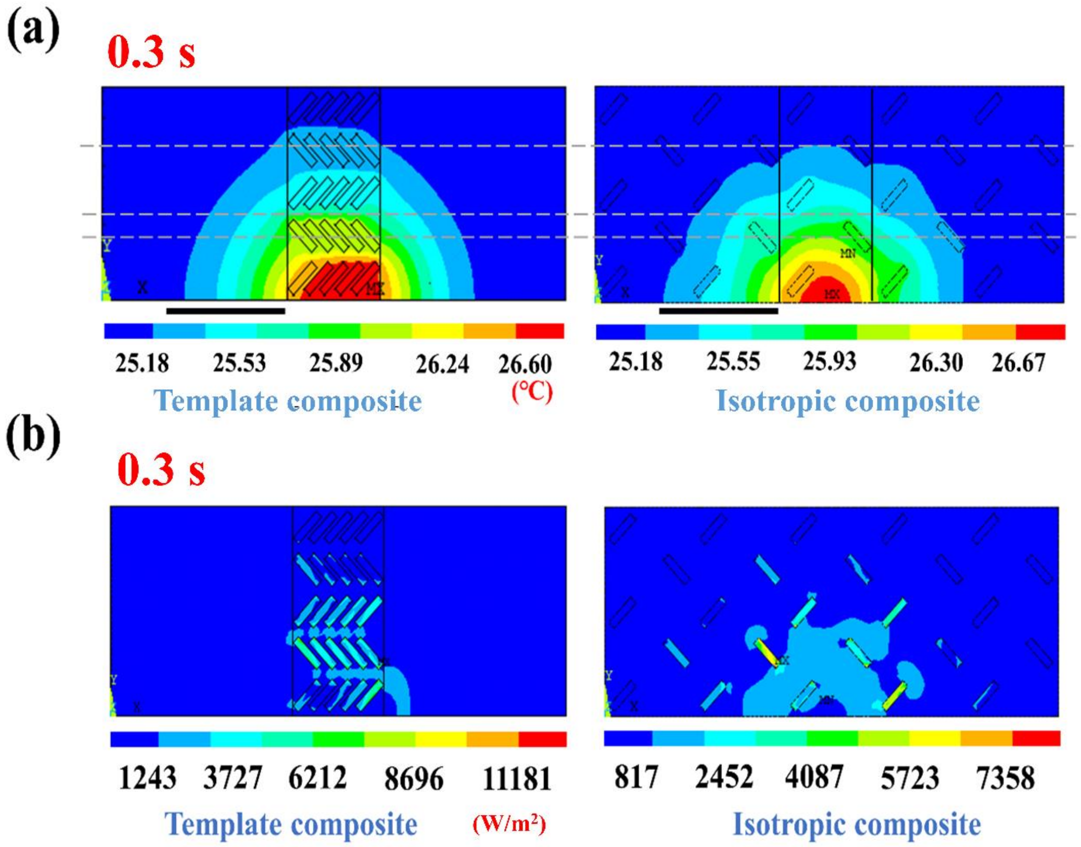

Figure S5. (a) Temperature contours and (b) sum of heat flux of different models at 0.3 s.

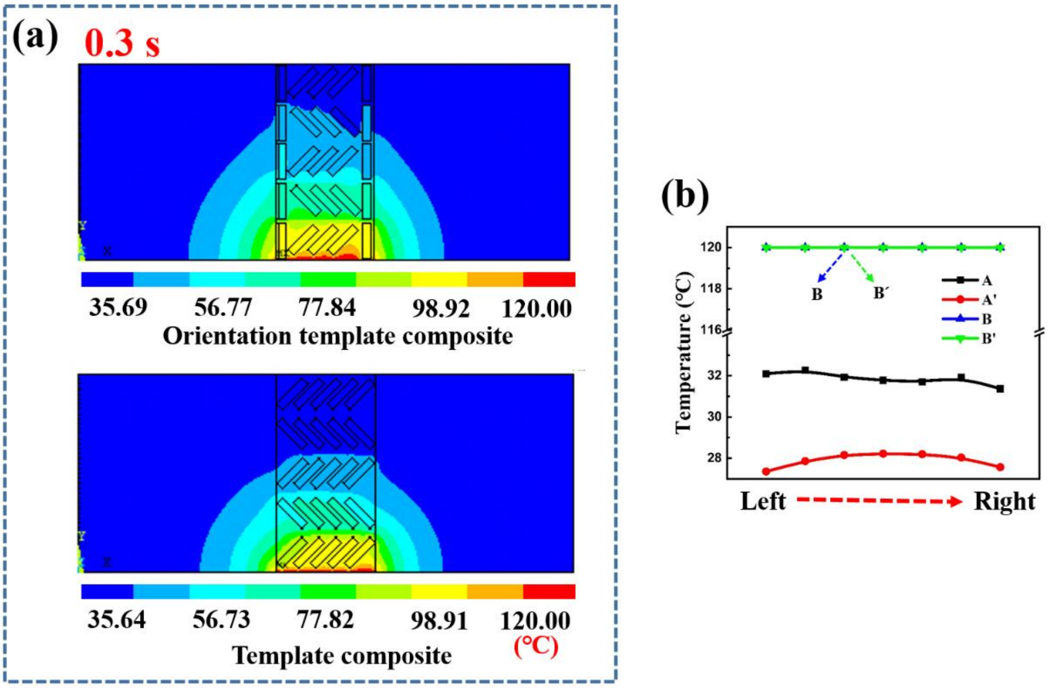

Figure S6. Temperature contours and temperature curves of different models with the constant heat source.

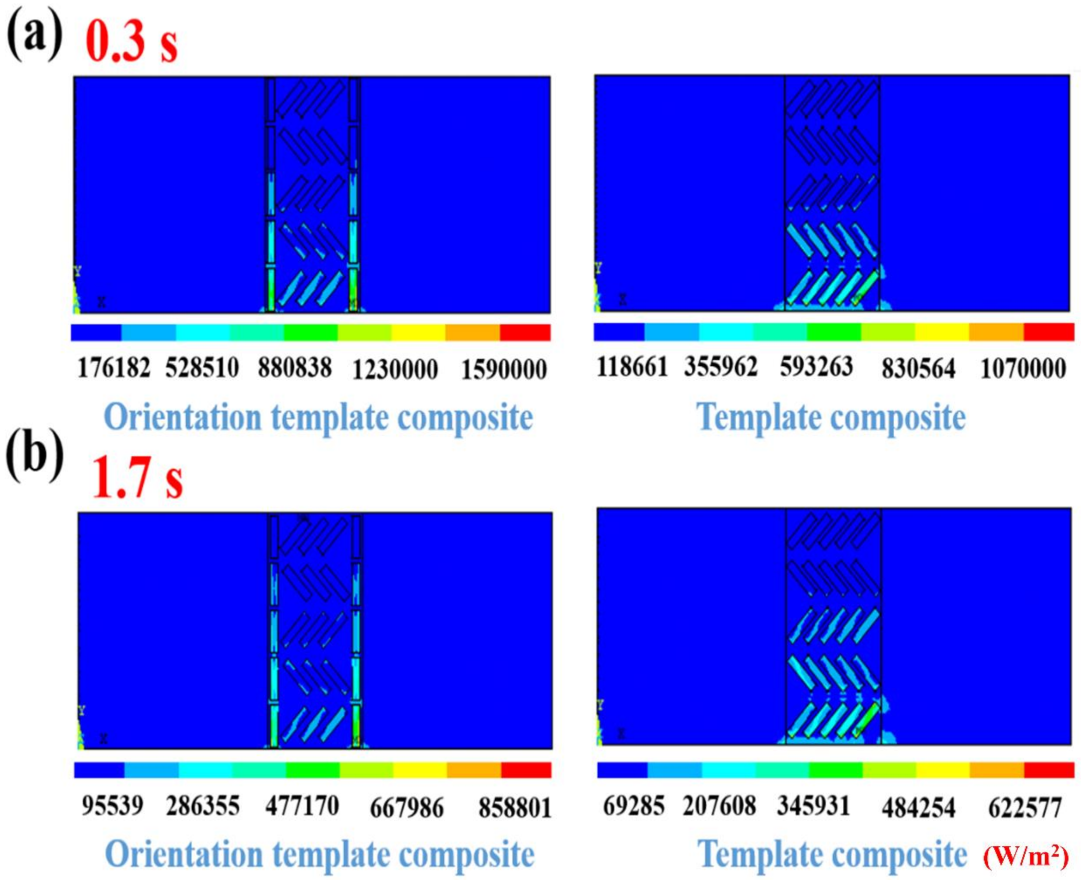

Figure S7. Sum of heat flux of different models loaded with the constant heat source at (a) 0.3 s and (b) 1.7 s.

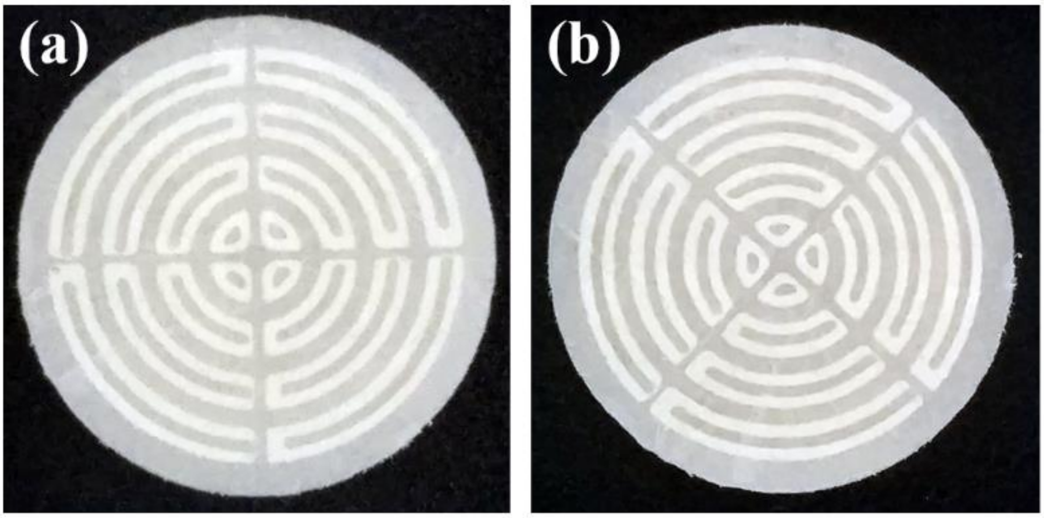

Figure S8. The optical images of the orientation template composite (a) before and (b) after the dropping test.

Mechanical properties of the orientation template composite:

Mechanical properties, such as elongation at break, tensile strength and so on, are important parameters for the practical application of the orientation template composite. However, due to the restriction of the sample size and structure, the mechanical properties of the orientation template composite cannot be measured directly. According to the testing method of electronic devices, the 1.5 m free-fall experiment was applied to evaluate the mechanical properties of the orientation template composite. The results are shown in **Figure** S8, it can be seen that the orientation template composite was not damaged in the test. Therefore, the orientation template composites have sufficient mechanical properties to meet the requirements of the thermal management applications in electronic devices.
